# Current and future suitability of wintering grounds for a long-distance migratory raptor

**DOI:** 10.1038/s41598-017-08753-w

**Published:** 2017-08-18

**Authors:** Christina Kassara, Laura Gangoso, Ugo Mellone, Gvido Piasevoli, Thomas G. Hadjikyriakou, Nikos Tsiopelas, Sinos Giokas, Pascual López-López, Vicente Urios, Jordi Figuerola, Rafa Silva, Willem Bouten, Alexander N. G. Kirschel, Munir Z. Virani, Wolfgang Fiedler, Peter Berthold, Marion Gschweng

**Affiliations:** 1 Department of Biology, University of Patras, GR-26500 Patras, Greece; 2 Department of Wetland Ecology, Estación Biológica de Doñana, CSIC, 41092 Seville, Spain; 3 Vertebrates Zoology Research Group, CIBIO Research Inst., University of Alicante, ES-03690 San Vicente del Raspeig, Alicante Spain; 4 Public Institute for the Protected Natural Values Management in the County of Split and Dalmatia, Prilaz braće Kaliterna 10, HR-21000 Split, Croatia; 5 Department of Biological Sciences, University of Cyprus, 1678 Nicosia, Cyprus; 6 Hellenic Ornithological Society, Themistokleous str. 80, 10681 Athens, Greece; 7 Cavanilles Institute of Biodiversity and Evolutionary Biology, University of Valencia, C/Catedrático José Beltrán 2, ES-46980 Paterna, Valencia Spain; 8 Computational Geo-Ecology Institute for Biodiversity and Ecosystem Dynamics, University of Amsterdam, 1098 XH Amsterdam, The Netherlands; 9 The Peregrine Fund, 5668 West Flying Hawk Lane, Boise, Idaho 83709 USA; 10 Max Planck Institute for Ornithology, Am Obstberg 1, D-78315 Radolfzell, Germany; 11 Institute of Experimental Ecology, University of Ulm, Albert-Einstein-Allee 11, D-89069 Ulm, Germany; 12 Concepts for Conservation, Schäferweg 6, 89143 Blaubeuren, Germany

## Abstract

Conservation of migratory species faces the challenge of understanding the ecological requirements of individuals living in two geographically separated regions. In some cases, the entire population of widely distributed species congregates at relatively small wintering areas and hence, these areas become a priority for the species’ conservation. Satellite telemetry allows fine tracking of animal movements and distribution in those less known, often remote areas. Through integrating satellite and GPS data from five separated populations comprising most of the breeding range, we created a wide habitat suitability model for the Eleonora’s falcon on its wintering grounds in Madagascar. On this basis, we further investigated, for the first time, the impact of climate change on the future suitability of the species’ wintering areas. Eleonora’s falcons are mainly distributed in the north and along the east of Madagascar, exhibiting strong site fidelity over years. The current species’ distribution pattern is associated with climatic factors, which are likely related to food availability. The extent of suitable areas for Eleonora’s falcon is expected to increase in the future. The integration of habitat use information and climatic projections may provide insights on the consequences of global environmental changes for the long-term persistence of migratory species populations.

## Introduction

Understanding how organisms interact with their environment and, especially, how they cope with environmental variability is a fundamental question in ecology and of critical importance to the conservation of migratory animals. Migratory species occur in geographically distinct areas during their annual cycle and exploit a variety of habitats^1^. However, because of the inaccessibility and remoteness of certain areas, the volatility of some political regimes and/or the high cost of large-scale field expeditions, researchers have often been restricted to the study of just a small subset of migratory species’ life stages, usually the breeding period. On top of this, the varying ecological needs of these species constitute a fascinating, yet challenging field of study, which has recently become even more complex in the context of rapid changes occurring at a global level.

The impact of aberrant weather conditions on the abundance and distribution of species has been recognized early during the past century^2–4^. More recently, the analysis of long-term data has provided sound evidence on the impact of climate change on living organisms. For instance, many birds have shifted the timing of breeding and/or migration in response to climate change^5, 6^. These changes may have cascading effects, often affecting food availability^7^, thus impacting breeding output and survival rates^8–11^, as well as triggering range shifts^12–14^. Even though climate will undoubtedly continue to be a key driver of species’ abundance and distribution in the future, anthropogenic habitat changes can incur equally severe effects on species’ ranges and fitness. For example, intensification of agricultural practices in Mediterranean countries have negatively impacted farmland birds’ populations and have been linked to lower adult survival- and population growth rates e.g. ref. ^15^ as well as to alterations in the species’ breeding distributions e.g. ref. ^16^.

Due to the strong endogenous control of migratory behavior, long-distance migrants are expected to be less resilient to the effects of global change^17, 18^, especially those species depending on seasonal habitats^19, 20^. Taking into account the velocity at which these changes occur as well as the complexity of developing appropriate management policies, the conservation of long-distance migrant species calls for prompt, high quality databased decision making processes to improve their future prospects e.g. ref. ^20^. Telemetry has recently allowed almost continuous remote tracking of animal movements providing opportunities to investigate the effect of natural and human-induced factors, including climate change, throughout their life cycle. Telemetry data may be coupled with future climate predictions and ecological niche modeling, thus allowing identifying key limiting environmental factors^21^, potential wintering areas^22–25^ as well as aiding the development of management guidelines^26^.

The Eleonora’s falcon (*Falco eleonorae*) is a long-distance migratory raptor that breeds in the Mediterranean basin and winters primarily in Madagascar^27, 28^, one of the eight ‘hottest’ biodiversity hotspots in the world^29^. The species was successfully tracked to Madagascar for the first time in 2003^28^. Since then, further studies analyzed the species’ flight characteristics and strategies during migration^30–33^, and its wintering habitat distribution^34–36^. During winter, Eleonora’s falcons are mainly insectivorous^37–39^ and food availability strongly depends on the landscape structure and environmental conditions^40–44^.

As any long-distance migratory species, Eleonora’s falcon may be affected by selective pressures and factors operating in widely separated geographically breeding and non-breeding areas, as well as during migration. This calls for an integrated inter-continent conservation program^45^, whose effectiveness largely depends on the availability of accurate information about the limiting factors throughout the range of habitats used by the species. Knowledge about the main non-natural factors threatening the persistence of the Eleonora’s falcon breeding populations is accumulating (e.g. persecution^46^, human disturbance associated with tourism development^47, 48^, and introduction of mammal predators^49, 50^. However, the identification of the main threats at its wintering grounds is still difficult to account for, due to the lack of precise information about its distribution and habitat use in Madagascar. Malagasy economy and subsistence relies heavily on the exploitation of natural resources, mainly through agricultural practices, which often result in severe habitat transformations. Consequently, the description of the Eleonora’s falcon habitat requirements and the timely prediction of the impact of global change on the suitability of its wintering grounds are considered essential tools for policy makers, who could then prioritize conservation measures at a global level.

Here, we determined habitat use by Eleonora’s falcons in Madagascar based on all available satellite and GPS tracking data from individuals breeding on the Canaries, Columbretes and Balearic islands (all in Spain), as well as in Italy, Croatia, Greece, and Cyprus^32, 34–36, 51^ (Gangoso *et al*. unpublished data, Hadjikyriakou *et al*. unpublished data). In spite of recent large-scale, multipopulation studies^52, 53^, to our knowledge this is the first time to study a highly mobile raptor species with data from individuals of the entire breeding range. We first explored whether habitat use patterns were consistent across years, as well as among individuals of different breeding origin. Based on these results, we proceeded to develop Species Distribution Models (SDM), which accounted for the effect of individual variability and annual heterogeneities in environmental factors. Finally, we assessed how the present habitat suitability for Eleonora’s falcon in Madagascar is likely to change based on future climate scenarios. Our ultimate goal is to provide a baseline framework for the Eleonora’s falcon conservation for the wintering area, where almost the entire population spends the majority of its annual cycle^27^.

## Results

### Exploratory space-use analyses

**Figure 1:**
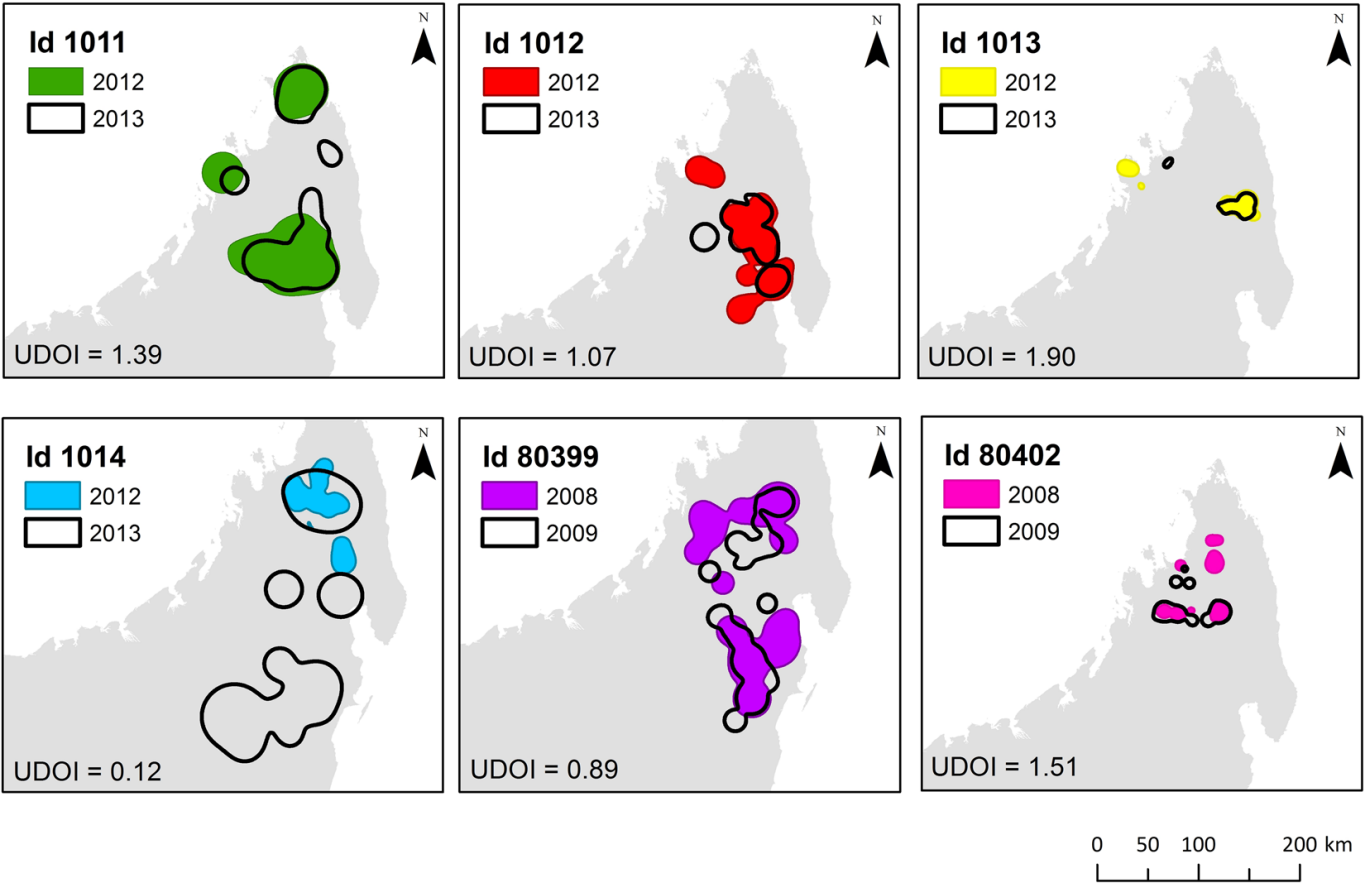
Strong winter site-fidelity for 6 Eleonora’s falcons as indicated by the overlap of their home ranges in consecutive wintering events and the corresponding value of the Utilization Distribution Overlap Index. Map was created with ArcGIS v10.1 (www.arcgis.com).

Space-use analyses demonstrated a high within-individual home range overlap between consecutive years (UDOI = 1.145 ± 0.614, N = 6 adults), suggesting strong winter site-fidelity (Fig. 1). This result allowed us to compare individuals tracked in different years in order to detect differences in home range overlap linked to breeding origin. The falcons’ breeding population did not have a significant effect on the overlap of the estimated home ranges (Table 1). In fact the inter-colony overlap was equal or even exceeded in some cases the intra-colony overlap (Supplementary Table S3), suggesting that falcons from different colonies may aggregate during the wintering period and exploit the same areas.

**Table 1:** Home range overlap analysis results based on 95% Kernel Density Estimation (KDE) of 23 Eleonora’s falcons overwintering in Madagascar analyzed using the Utilization Distribution Overlap Index (UDOI).

Group	UDOI	Compared groups	Mann-Whitney U (p value)
Akrotiri (Cyprus)	0.000 (N = 1)	Cyclades vs Sardinia	45.000 (p = 1.000)
Cyclades (Greece)	0.029 (N = 6)	Cyclades vs Canary Islands	15.000 (p = 0.630)
Svetac (Croatia)	0.000 (N = 1)	Sardinia vs Canary Islands	35.000 (p = 0.431)
Sardinia (Italy)	0.065 (N = 15)	Adults vs Juveniles	396.000* (p = 0.019)
Balearics (Spain)	0.021 (N = 1)		
Columbretes (Spain)	0.000 (N = 1)		
Canary Islands (Spain)	0.035 (N = 6)		
Adults	0.041 (N = 139)		
Juveniles	0.009 (N = 10)		

Statistical significant differences are annotated with an asterisk (*). N denotes the number of inter-individual UDOI values averaged per colony and age class (group).

**Figure 2:**
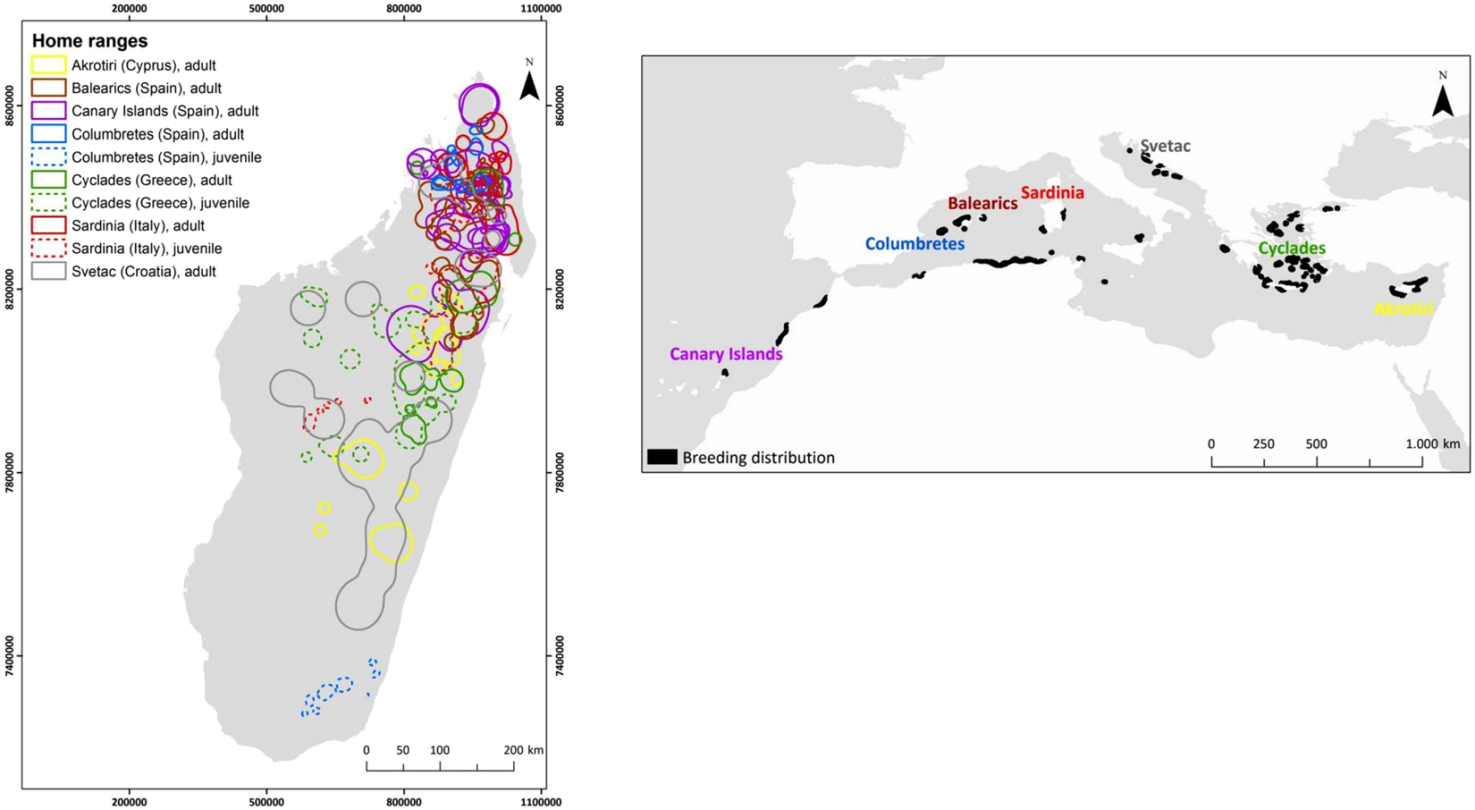
Home ranges of 23 Eleonora’s falcons marked in colonies spanning from the westernmost (Canaries) to the easternmost (Cyprus) breeding range of the species. The existing breeding colonies are highlighted in black (data from^87^). Map was created with ArcGIS v10.1 (www.arcgis.com).

The subsequent comparisons indicated a strong age effect; the juvenile overlap was significantly lower than adult overlap (Table 1). Accordingly, the distribution patterns of the 6 juveniles differed considerably among them, as well as compared to the adults. While adults were mainly distributed in the north and central part of Madagascar, some juveniles roamed also in the southern and western part of the country (Fig. 2). Nonetheless, considering the small sample size of juveniles (N = 6), as well as the fact that first calendar-year falcons are expected to be more prospective and have less defined habitat preferences than adults, we used only adult data (N = 17) to model habitat suitability for Eleonora’s falcon in Madagascar.

### Present habitat suitability

**Figure 3:**
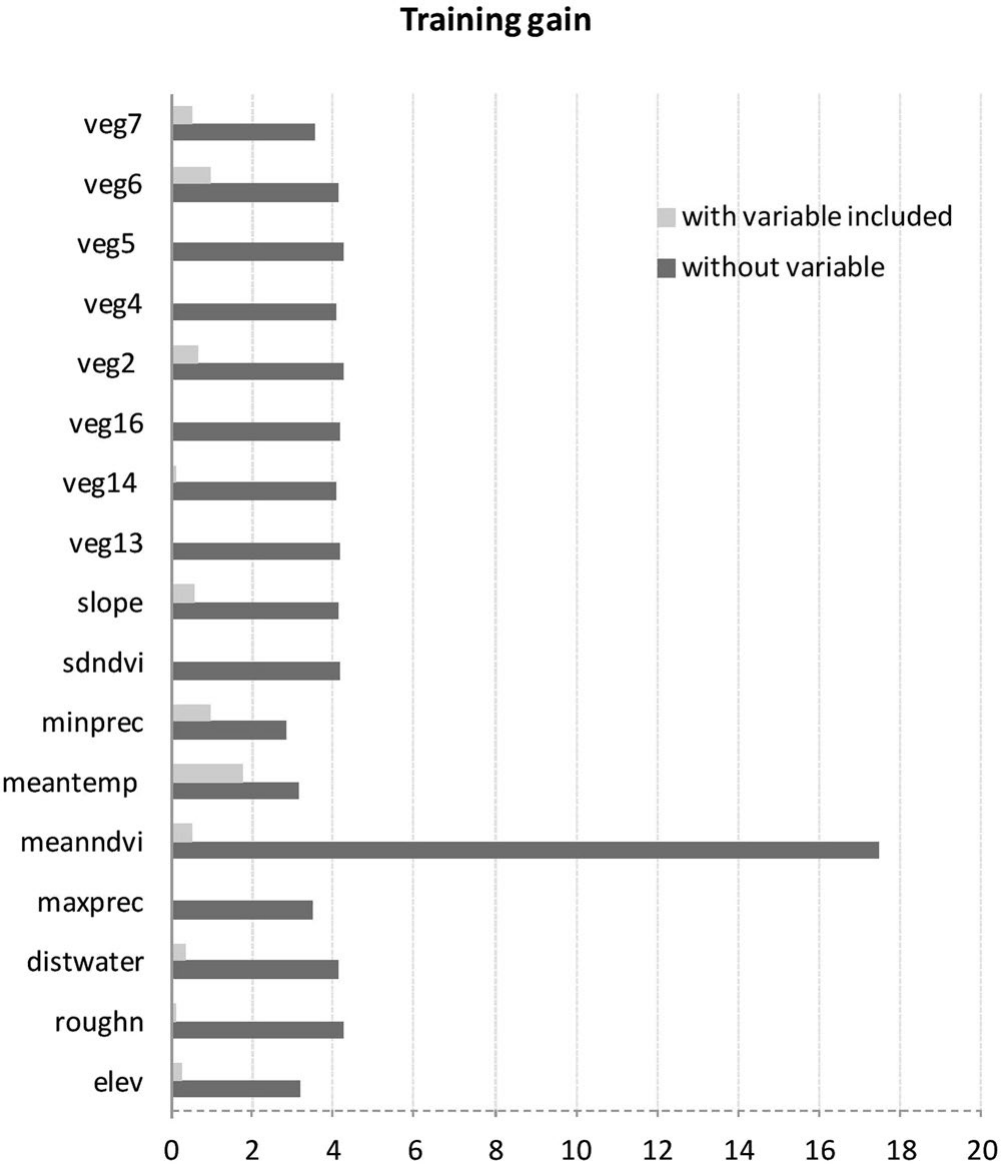
Training gain results (average of 100 models based on adult data only) for the explanatory variables used in model building. According to these, the most significant predictors of habitat suitability during the wintering period are minimum precipitation (minprec) and mean maximum temperature (meantemp). Elevation (elev) is also considered to be an important predictor of habitat suitability, since it was found highly correlated with mean maximum temperature and thus its effect might have been overshadowed by the latter. Variable abbreviations as in Supplementary Table S2.

**Figure 4:**
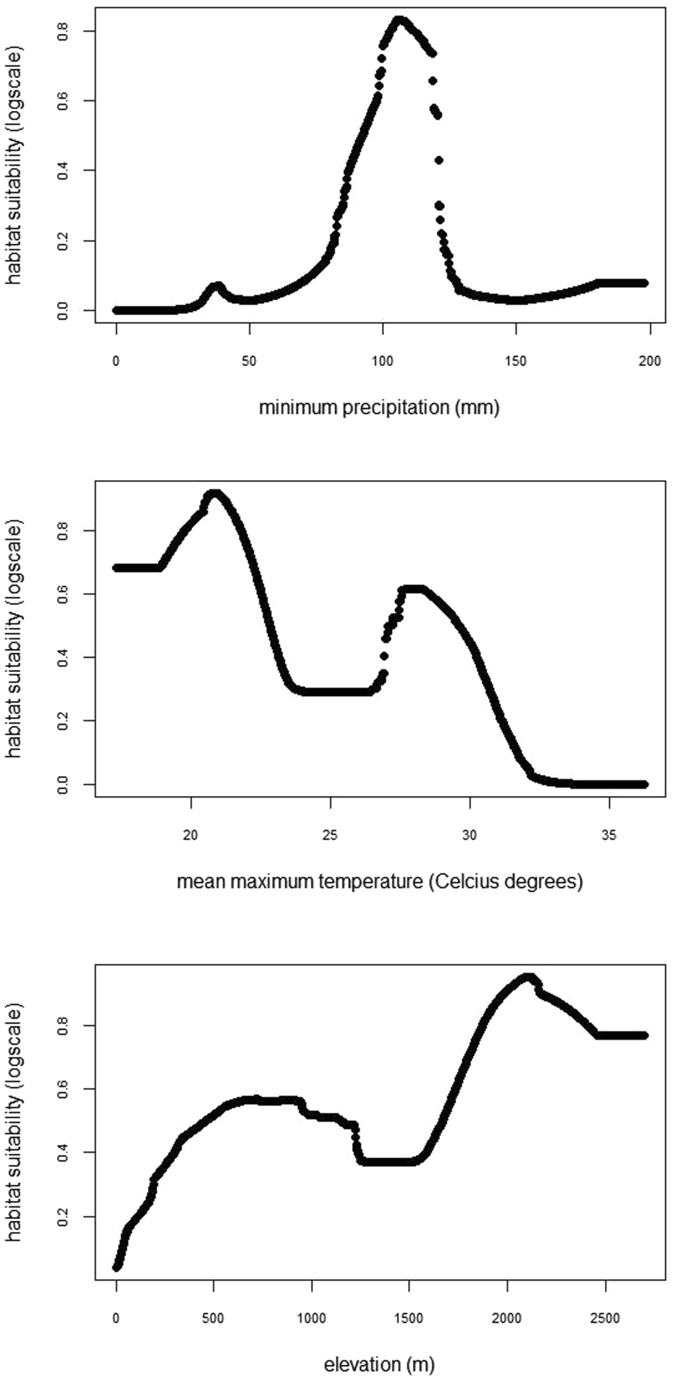
Response curves (average values of 100 models based on adult data only) of the three most significant predictors of habitat suitability for Eleonora’s falcon during the wintering period.

**Figure 5:**
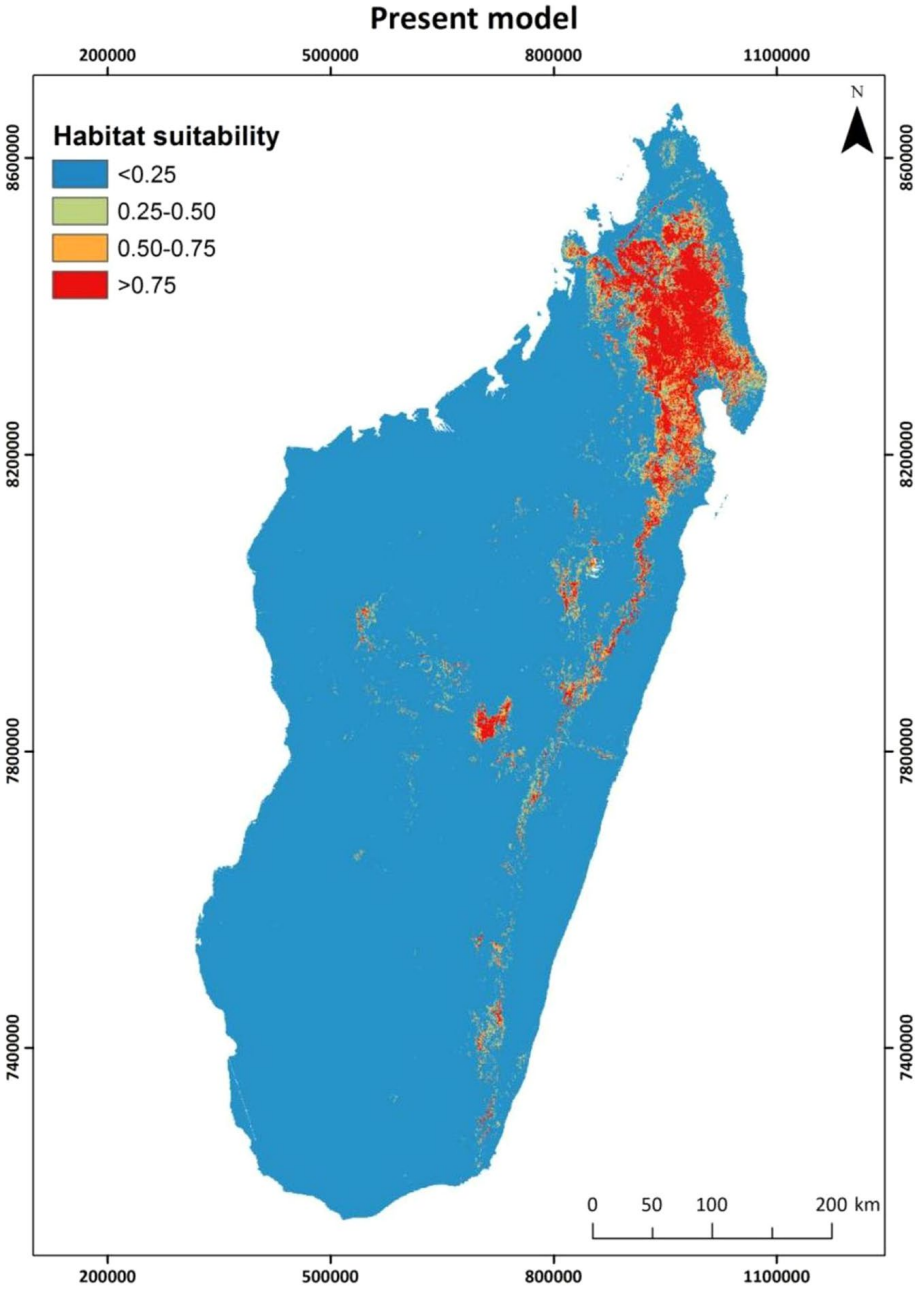
Predicted habitat suitability (average of 100 models) according to current environmental conditions for Eleonora’s falcon in Madagascar, based on satellite telemetry data of 17 adult falcons originating from colonies spanning from the westernmost (Canaries) to the easternmost (Cyprus) breeding range. Map was created with ArcGIS v10.1 (www.arcgis.com).

According to the Maxent model results, minimum precipitation and mean maximum temperature were the main factors related to Eleonora’s falcon wintering habitat distribution (Fig. 3). Given the fact that mean maximum temperature is highly correlated with elevation (*r* = 0.84, *p* < *0*.*001*) as well as the drop in training gain when elevation was omitted from the model (Fig. 3), we also consider the latter an important determinant of the species’ habitat suitability. Therefore, according to the corresponding response curves (Fig. 4), habitat suitability peaks in areas around 2100 m, receiving in total at least 100–120 mm of rainfall during the wintering period, where maximum temperature does not exceed 20–22 °C. The areas fulfilling these criteria are mainly located in the northern part of Madagascar, the Tsaratana Massif region, as well as along the eastern part of the country and central highlands (Fig. 5).

The models fitted the training datasets well (training AUC = 0.960 ± 0.004) and their predictive power was high (test AUC = 0.953 ± 0.006). In addition, the fit of the models was statistically significant compared to null models (training AUC of null models = 0.540 ± 0.017, 95% confidence limits of null models = 0.538, 0.541).

### Future habitat suitability

**Figure 6:**
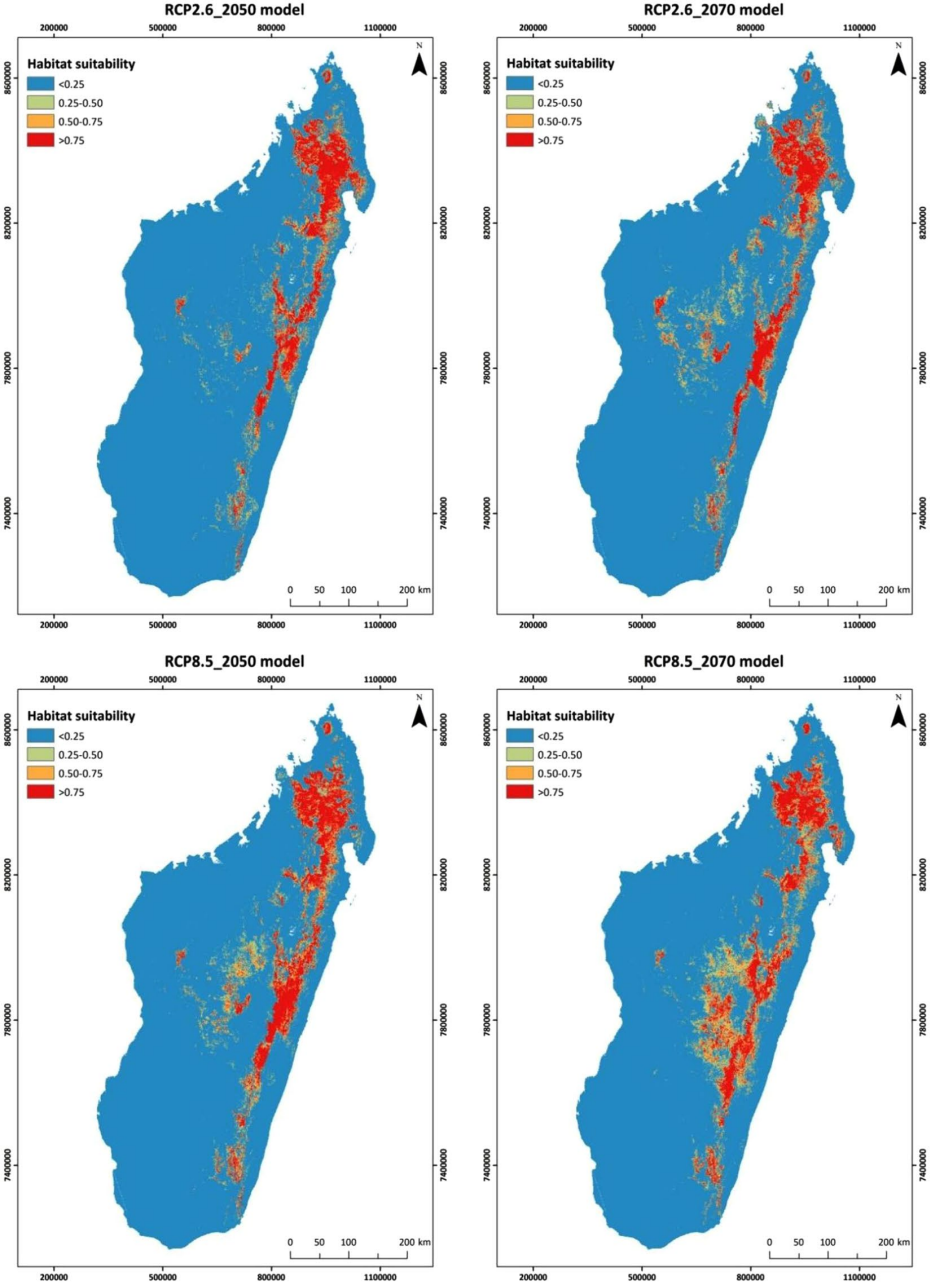
Predicted habitat suitability according to future climate scenarios (RCP2.6 above and RCP8.5 below) for 2050 (left) and 2070 (right) for Eleonora’s falcon at its main wintering area, Madagascar, based on satellite telemetry data of 17 adult falcons. Map was created with ArcGIS v10.1 (www.arcgis.com).

Compared to present conditions, the extent of suitable areas for Eleonora’s falcon is expected to increase in the future. For instance, considering those areas with a habitat suitability score of at least 0.75 as most suitable, then under present climatic conditions such appropriate areas cover a surface of 28,346 sq. km. In the future, however, they will extend over an area between 38,697 and 44,710 sq. km., according to the projected year and climate scenario (Table S4). In addition, our results suggest a southward shift in the location of the most suitable habitat, a reduction of highly suitable areas in the North and an increase along the center and East of Madagascar extending further southwards (Fig. 6), which is dominated by evergreen humid forests^54^ and croplands.

## Discussion

The Eleonora’s falcon is one of 22 raptor species in the world that are complete migrants at a global level^55^. Owed to its large breeding range and (apparently) increasing population, estimated in 29,200–29,600 mature individuals, the Eleonora’s falcon is classified as Least Concern^56^. Nonetheless, it is unevenly distributed within its breeding range, with Greece holding more than 85% of the global population^57^, while the increase in population numbers observed in late years is attributed to more extensive field surveys^57^ rather than to an actual increase. The Eleonora’s falcon is well-known for delaying its breeding season to late summer to take advantage of the autumn flux of small migratory birds^27^. Nonetheless, this strong linkage to this particular food source during the breeding period could seriously challenge its persistence, given the already evidenced impacts of climate change on the phenology of passerine migration^58^. In the present study, we showed that adult Eleonora’s falcons are faithful to a common, relatively small wintering area, apparently forming a single wintering population regardless of their breeding origin. Therefore, the selective pressures, such as those resulting from climate change, faced in this restricted area are expected to have a more severe impact on the species’ persistence, given that they would affect the whole Eleonora’s falcon population, compared to the unequal influence of local factors acting across the species’ breeding range^46–50^.

Our previous research highlighted the dominant role of climate, topography and habitat type in the wintering habitat distribution of a few individuals from breeding colonies in Spain^35^, Italy^34^ and Greece^36^. In the present study we were able to generate a species-wide wintering habitat suitability map for the Eleonora’s falcon, by analyzing 23 wintering events spanning a decade and pertaining to 17 adults from 7 breeding colonies. To our knowledge, this is the first time that a habitat suitability model has been generated for a species based on data from individuals originating from colonies across its entire breeding distribution. Therefore, our habitat modeling approach provides a solid benchmark for the species’ conservation and will help answer critical questions about their resilience to global environmental changes.

### Present habitat suitability

Our results indicated that habitat suitability for the Eleonora’s falcon in Madagascar is mainly shaped by regional variations in climate regime, in particular precipitation and temperature, both likely related to insect prey availability^37–39^. Beyond the variety of factors influencing insect abundance at various spatial and temporal scales, climatic variables, such as temperature and humidity, are considered among the key drivers of insect abundance and phenology^41, 43, 44^. Tropical insect species tend to peak in numbers during the wet and transitional seasons^59^ (and references therein), given the time lag in vegetation phenology^42, 60^. Insects abound in areas dominated by humid forest and cultivations characterized by moderate to high precipitation levels^42^, such as the northern and central highlands of Madagascar. At the same time, woodlands with openings attract a high number of insects^61, 62^. Such landscape mosaics in Madagascar are the result of extensive land use changes since the 1970s, where a large part of forests (both humid and dry spiny forest) have been cleared out for cultivations^63, 64^. Compared to our previous findings^35, 36^, the lack of a direct effect of the percent cover of humid and/or degraded forests (namely, veg 14 and 16) on habitat suitability does not undermine the ecological significance of these habitat types for Eleonora’s falcon, but is rather linked to the fact that they occur in relatively fragmented and isolated patches^36^. Thus, although the ecological drivers of Eleonora’s falcon habitat suitability in Madagascar, namely precipitation, temperature and elevation, are consistent with the species’ food requirements, it is foreseeable that the ongoing forest felling and increased use of biocides can limit the availability of food for Eleonora’s falcons and ecologically related species. This raises an interesting conservation debate; while the increase in degraded land may favor Eleonora’s falcons in the short term, since they often use cultivated areas and degraded forests to forage^35, 36^, the massive habitat loss undoubtedly poses a serious threat for the entire biodiversity of Madagascar and, in the long term, for Eleonora’s falcons too.

Adult Eleonora’s falcons showed a high degree of site fidelity among years, yet they use multiple core areas likely associated with temporal and regional variations in rainfall patterns. On the contrary, juveniles were more prospective than adults, exploring a larger part of the country and, thus exhibiting a more diffuse distribution pattern (Fig. 2). Still, although the reduced sample size precluded further analyses, the distribution pattern of the 6 juveniles indicated that the most frequented regions were wetlands and areas with minimum precipitation levels above 100 mm, similar to our findings for adult falcons, occurring at areas with at least 100–120 mm of rainfall.

### Future habitat suitability and implications for conservation

Our predictions indicate that the extent of suitable habitat for Eleonora’s falcon in Madagascar will increase over the next 50–60 years, even under the worst climate scenario (RCP8.5: high emissions scenario). More specifically, current suitable habitats in the north will be reduced, while habitats located along the eastern and central parts of the country, dominated by humid forests, cultivated areas and grasslands, will become more favorable for the species. However, the most vulnerable regions to climate change in the East are considered to be the ones lying from 1200 to 1600 m altitude^65^, which partially coincide with highly suitable areas for the species under the worst climate change scenario (namely, RCP 8.5 2070).

Agroecosystem responses to global environmental changes, including the insect community, are harder to predict, given the intensive human-related habitat transformations. Considering that deforestation is still ongoing in Madagascar, further habitat loss may counterbalance the potential positive effects of more favorable climatic conditions. While the northern highlands have remained fairly intact due to their inaccessibility, the forests lying in the central and southern parts of the country, which are expected to be rendered suitable for Eleonora’s falcon in the future, can be largely impacted by the expansion of agricultural practices. Thus, the predicted southwards shift of the species’ habitat suitability and its expansion into cultivated areas in the central part of Madagascar could result in an increased exposure of the species to anthropogenic habitats and to the biocides used in agriculture, reducing prey availability and increasing the risk of secondary poisoning^66^.

Consequently, although our predictive models may suggest optimism with regards to future availability of suitable wintering habitat, our results should be considered as a possible outcome all other things being equal. Moreover, the species is an island dweller both during its breeding and wintering period and a highly specialized raptor in terms of food requirements^37^. In this regard, specialist predators are predicted to have a lower potential to buffer environmental variations than generalist species^67, 68^. In addition, Eleonora’s falcon population gathers at a relatively small wintering area (present and predicted) relative to the size of the country. Therefore, strong perturbations in Madagascar associated with extreme weather events, habitat loss and decline in habitat quality (mainly as a result of human intervention), may induce carry-over effects, affecting species’ long-term fitness^69^. Other factors fuelled by global environmental change, such as the use of biocides, should be considered an issue of major concern for the species’ conservation. For example, following the locust outbreak in 2012 in Madagascar, the widespread use of insecticides (more than 42,000 km^2^ were sprayed within four years) caused serious declines in populations of non-target invertebrates and vertebrates, leading to food-chain perturbations^70^. Such outbreaks are likely to increase as a consequence of climate change^65^, raising serious concerns about mitigation measures-derived hazards to wildlife.

Moreover, the highly suitable areas (i.e. those with a habitat suitability score over 0.75) under current conditions overlap spatially with the corresponding areas under future conditions at just 59.54% (SD = 6.07%), suggesting that the Eleonora’s falcon current distribution will change substantially in the following years. On top of this, the existing protected areas network in Madagascar overlaps only a fraction (less than 20%) of the highly suitable habitat for the Eleonora’s falcon both under present and future climatic conditions (see Supplementary Table S5 and Fig. S2). Therefore, our model predictions may on one hand help to identify the key areas for this falcon in the future in order to develop appropriate conservation strategies. On the other hand, our results can contribute to the reinforcement of the protection status of the habitats Eleonora’s falcon depends upon, especially primary evergreen forests, and thus the biodiversity these habitats host as a whole.

To sum up, the natural landscape of the Eleonora’s falcons’ wintering area has been altered by drastic human intervention in a very short period of time and is expected to be largely affected by climate change in the future^37, 71, 72^. Regardless of the uncertainty surrounding future climate scenarios, as well as the species’ ability to disperse, colonize and survive in the newly climatically suitable areas, our results provide a compass for future studies furnishing valuable information for the species’ conservation in Madagascar. More specifically, we explored habitat use patterns and winter site-fidelity through home range analysis and created a species-wide habitat suitability model, providing the first evidence of climate change effects on habitat suitability. Given the increased pace of climate change, and in alignment with the current trend in animal ecology and conservation, we prompt future studies to investigate to what extent genetic variation, plasticity and population dynamics jointly determine how migratory species living in seasonal environments may respond to the ongoing and future environmental changes. This multidisciplinary approach will contribute to the re-evaluation of conservation priorities at a global level, a task that should reconcile trade-offs between biodiversity conservation and socioeconomic development in a framework of unprecedented global change.

## Methods

Eleonora’s falcon present and future habitat suitability was modeled using MaxEnt, one of the most powerful SDM methods^73, 74^. MaxEnt is a niche-modeling technique for presence-only data, which approximates habitat suitability by identifying areas that share the same environmental conditions (i.e. combination of ecological variables) as those areas where the target species has been recorded with a given set of environmental variables. In order to identify which environmental conditions are mostly preferred by the target species, MaxEnt contrasts the values of the candidate environmental variables between the area occupied by the species and the available geographical space, known as “background”.

Presence-background models, like MaxEnt, that are based on satellite telemetry data, especially when derived from a large number of individuals, can provide robust predictions on the geographic distribution of more suitable environmental conditions at the species-level^75^. In this context, our methodological approach is intended to produce a reliable species-wide habitat suitability map under present and future environmental conditions.

Data preparation was implemented in ArcGIS v10.1^76^ statistical analyses were performed in R v3.2.2^77^, while SDMs were carried out in MaxEnt v3.3.3k^78^. Details on capture methods and animal handling have been described in our previous papers^34–36^. The methods described here have been adapted from^34^ and^36^. A detailed description of methods used is provided in the Supplementary material. Mean values and standard deviations are reported. Statistical significance level was set to α = 0.05. All spatial data used for the subsequent analyses, as well as the maps presented herein have been projected to WGS 84/UTM zone 38 S (EPSG: 29738).

### Occurrences

We compiled all available telemetry data to date (i.e., from 2003 to 2014) for the wintering period (November–April) of Eleonora’s falcon in Madagascar. These telemetry data derived from 23 individuals (adults and first calendar year juveniles, hereafter “juveniles”, Table 2) originating from colonies located across the entire species’ breeding range and adding to 30 wintering events. Following appropriate filtering (see Supplementary Appendix 1) c.a. 5,000 data points constituted the data pool for subsequent analyses (Supplementary Table S1).

**Table 2:** Summary of satellite telemetry data for Eleonora’s falcon at its Malagassy wintering areas used in this study.

Colony	Wintering period	Number of tracked individuals	
		Adults (*)	Juveniles
Akrotiri (Cyprus)	2013–2014	2	—
Cyclades (Greece)	2009–2010	2	2
Svetac (Croatia)	2009–2010, 2010–2011	2	—
Sardinia (Italy)	2003–2004, 2004–2005, 2005–2006	4	3
Balearics (Spain)	2008–2009, 2009–2010	2 (1)	—
Columbretes (Spain)	2008–2009, 2009–2010	1 (1)	1
Canary Islands (Spain)	2012–2013, 2013–2014	4 (4)	—
**TOTAL**		**17 (6)**	**6**

*Number of individuals for which data were available from two consecutive wintering events and which fed the winter site-fidelity analyses.

### Environmental correlates

According to the species’ ecology and the results of our previous research^34–36^, we considered topography (elevation, topographic roughness), proximity to fresh water bodies, vegetation composition (percentage of vegetation classes), vegetation phenology (NDVI) and climate regime (mean maximum temperature, maximum and minimum precipitation) as candidate predictors of habitat suitability for the occurrence of Eleonora’s falcon at its wintering quarters (Supplementary Table S2).

#### Exploratory space-use analyses

Prior to model building we assessed whether patterns of habitat use differ during the wintering season among years, populations and age classes. In particular, we estimated home range (95%-KDE) utilization distribution (UD) for each individual and wintering event based on the aforementioned occurrence data pool (see Supplementary Appendix 1). Firstly, we assessed wintering site-fidelity by calculating the UD Overlap Index (UDOI^79^), for the same individual between consecutive wintering seasons (N = 6 individuals). We then calculated UDOI between age classes and populations. To avoid pseudo-replication, we averaged the UDOI values derived from multiple wintering events referring to the same individual.

#### Modeling present habitat suitability

According to the results of the space-use analyses, we generated habitat suitability models considering adults separately following the procedure outlined below (for a detailed description please see Supplementary Appendix 1).

In order to achieve equal representation of all individuals during model building^80^, we created 10 random subsamples for each individual that equaled the number of data points of the falcon with the smallest sample size rounded to the nearest integer (Supplementary Table S1). Taking into account the number of explanatory variables^81^, we trained the MaxEnt model with 80% of each presence subsample and evaluated the model predictive performance with the remaining 20%. Data splitting was conducted 10 times at random per subsample. Overall, we created 100 models (10 subsamples for calibration × 10 subsamples for evaluation). Model results are reported as averages of these 100 MaxEnt models (hereafter, “**present model**”). The predicted habitat suitability scores range from 0 to 1 (i.e. logistic output, which approximates the probability of occurrence).

As a measure of the overall model’s predictive power, MaxEnt uses by default the Area Under the Curve (AUC score^82^). However, considering the criticism against its use as a metric of model accuracy, especially in large study areas as in our case^83^, we assessed the statistical significance of the AUC scores of the resulting 100 models by contrasting them with the resulting values derived from null models following the recommended approach by^84^.

In order to assess the predictive power of the candidate explanatory variables we compared the variance explained by each variable when used in isolation (i.e. resembling a univariate model) and the information lost when that variable was omitted from a model containing the remaining variables.

#### Modeling future habitat suitability

We predicted future habitat suitability based on different climate change scenarios, using the latest Global Climate Models (GCMs) of the fifth phase of the Coupled Model Intercomparison Project (CMIP5; http://cmip-pcmdi.llnl.gov/cmip5/). GCMs are generated based on scenarios concerning emissions of pollutants, future climatic and environmental conditions, as well as socioeconomic changes. CMIP5 in particular considers four scenarios, known as Representative Concentration Pathways (RCPs), of which we chose the two extremes, the low emissions scenario (RCP 2.6) and the high emissions scenario (RCP 8.5) to model future habitat suitability (for an overview of the RCPs see^85^). Among the available GCMs we used future climate data produced by the HadGEM2-ES model^86^ for 2050 and 2070. We generated four future habitat suitability projections (2 scenarios × 2 years; hereafter, “**RCP2**.**6_2050 model**”, “**RCP2**.**6_2070 model**”, “**RCP8**.**5_2050 model**”, “**RCP8**.**5_2070 model**”) by repeating the methodological steps described above for the present model.

## Electronic supplementary material

Supplementary material
