# Supplementary material for: Current and future suitability of wintering grounds for a long-distance migratory raptor
Source: Sci Rep. 2017 Aug 18;7:8798. doi: 10.1038/s41598-017-08753-w (PMC5562895; doi:10.1038/s41598-017-08753-w)
Supplement: Supplementary file 1 — Supplementary material [file 41598_2017_8753_MOESM1_ESM.doc]

Kassara, C., Gangoso, L., Mellone, U., Piasevoli, G., Hadjikyriakou, T. G., Tsiopelas, N., Giokas, S., López-López, P., Urios, V., Figuerola, J., Silva, R., Bouten, W., Kirschel, A. N. G., Virani, M. Z., Fiedler, W., Berthold, P. & Gschweng, M. Current and future suitability of wintering grounds for a long-distance migratory raptor.

**Appendix 1. Methods**

*Occurrences* - We compiled all available telemetry data to date (i.e. from 2003 to 2014) for the wintering period (November – April) of Eleonora’s falcon in Madagascar. These telemetry data derive from individuals captured and tagged from colonies located across the entire species’ breeding range, from the most eastern colony in Cyprus to the most western one at the Canary Islands1,2,3,4,Gangoso et al. unpublished data, Hadjikyriakou et al. unpublished data. Eleonora’s falcons were trapped during the breeding season either at their nest or using mist nets and luring and subsequently tagged with PTT or GPS devices (Table S1) following standard protocols. Satellite data collected via the Argos system (i.e., with the use of PTT tags) can vary in terms of positional accuracy. Thus, we retained only the ones of high positional accuracy (i.e., LCs 1-3) in order to minimize the location error, as well as to get comparable data with the ones retrieved via the GPS tags. In addition, given the extremely higher sampling intensity of GPS data in comparison to the PTT data, we chose randomly 4 GPS fixes per individual every third day for the subsequent analyses, which approximates a 12hr ON/ 58hr OFF duty cycle. This subsample represents quite well the true trajectory of individual birds, as evidenced by the comparison between this subsample and the original high resolution (5-min fix interval) sample (see below and Fig S1).

Contrary to our previous research, we included both day and night-time data points, since there is evidence that Eleonora’s falcons forage throughout the day, as well as partly during the night^2,5^. Hence, following the removal of spurious locations the initial datapool (hereafter, **“original dataset”**) consisted of 6,257 data points corresponding to 23 individuals (5 males, 15 females and 3 of unknown sex, of which 17 were adults and 6 were juveniles) and 30 wintering events (Table S1). We excluded data points received 7 days after the falcons’ arrival at Madagascar and 7 days prior to the onset of spring migration that could be attributed to migratory restlessness.

Spatiotemporal autocorrelation, either as a result of sampling bias or being an inherent property of tracking data, violates the assumption of independence of occurrence data in Species Distribution Modeling^6^. Spatial filtering has been typically used to account for sampling bias, although care should be taken to avoid ending up with too few occurrence data to build valid models^7^ or under-representing areas that are intensively used and thus constitute more suitable areas. In our case and given the species’ high mobility, we only considered consecutive data points that were received at least 1 hour apart and located at least 1km apart. Thus, the original dataset was reduced to 4,967 data points (Table S1), corresponding to 21 individuals (5 males, 14 females and 2 of unknown sex, of which 17 adults and 4 juveniles) and 27 wintering events (hereafter, “**modeling dataset**”). The modeling dataset was used for all subsequent analyses.

Exploratory space-use analyses

Since the modeling dataset comprised of data from different populations, age classes and tracking years, we first wanted to explore any effect on space use that could be attributed to these parameters. We thus estimated and compared the overlap between the home ranges of the individuals relative to the aforementioned parameters. In order to delineate the home range of each falcon, we estimated the utilization distribution (UD) of each individual and wintering event (N = 30) by computing bivariate normal kernel densities. The smoothing parameter h, shown to be a critical parameter for kernel analyses, was estimated by a fixed ad hoc choice in each case as recommended in^8^. Other methods for estimating h were less suitable. For instance, the reference bandwidth (h-ref) method clearly overestimated home range sizes in most cases (Fig S1 B), while Least Square Cross Validation (LSCV) method could not be computed in some cases due to convergence problems, especially when locations were are close to each other or when they show clumped distributions^9^, as in our case. The kernels resulting by means of the ad hoc method were almost identical when using the original high resolution dataset and the resampled dataset (Fig S1 C).

To evaluate site fidelity of Eleonora’s falcons between years (only available for adults) and space-use overlap between age-classes and populations, we estimated home range (95% KDE) utilization distribution (UD) overlap using the UD Overlap Index (UDOI^10^). The UDOI quantifies overlap based on the product of the utilization distributions of 2 samples. It generally ranges between 0 (no overlap) and 1 (100% uniform distribution), but it can be >1 if the UDs of the 2 samples are aggregated in space and show much overlap ^10^. We first calculated UDOIs for each individual and wintering season and compared intra-individual overlap indexes across years. Second, we calculated overlap between age classes and populations. For these analyses, we used the average UDOI for individuals tracked for two consecutive years (N=6), so that each bird was an independent unit.

All analyses were conducted in R with the package adehabitatHR^11^. Differences in overlap values between groups were assessed by means of Mann-Whitney test.

*Background data* - The irregular sampling originating from the duty cycle of the PTT devices could have masked out areas that had been actually used by the individuals, leading to omission errors. To this end, we restricted the choice of background points to 10,000 random points located within a Minimum Convex Hull polygon recreated from the original dataset of data points. To minimize the spatial autocorrelation among these background points, as well as to ensure that they represented true absence points given the high mobility of the species, the random background points were conditioned to be located at least 1km apart from each other and at least 10km away from the data points of the original dataset.

*Environmental correlates* - According to the species’ ecology and the results of our previous research^1,2,3^, we considered the topography (elevation, topographical roughness), proximity to water, vegetation composition (percentage of vegetation classes), vegetation phenology (NDVI) and climate regime (mean maximum temperature, maximum and minimum precipitation) as candidate predictors (hereafter, “explanatory variables”) of habitat suitability for the occurrence of Eleonora’s falcon at its wintering quarters (Table S2). To this end, we generated a grid of cells with a consistent resolution of 1 square kilometer across Madagascar and linked the 17 explanatory variables with each 1km^2^ grid square.

Prior to model building, we checked for the existence of multicollinearity among the explanatory variables, which could potentially overshadow the effect of a particular predictor^12^ by calculating Spearman’s correlation coefficient (*r*) between all possible pairs. Statistically significant high correlation occurred between elevation and mean maximum temperature (*r*=0.84, *p<0.001*). However, we decided to include both variables since they are ecologically relevant^12^.

Model present habitat suitability

Based on the results on the effect of tracking year, breeding origin and age class on space-use, we generated habitat suitability models utilizing the adult occurrences following the procedure outlined below.

In order to achieve equal representation of all individuals during model building ^13^, we created 10 random subsamples for each individual from the modeling dataset that equaled the number of data points of the falcon with the smallest sample size rounded to the nearest integer (i.e., 40 data points; Table S1). Taking into account the number of explanatory variables^14^, we trained the MaxEnt model with 80% of each presence subsample and evaluated its predictive performance with the remaining 20%. Data splitting was conducted 10 times at random per subsample. Thus, in total we created 100 models (10 subsamples for calibration x 10 subsamples for evaluation). We report model results as averages of the 100 MaxEnt models (hereafter, “**present** **model**”).

We ran MaxEnt under default settings, except that we set the regularization parameter (β) to 2 in order to reduce over-fitting given the number of tagged individuals used from each geographical area compared to the total local breeding population^15^. We chose the logistic output, which approximates the output of logistic regression; however, rather than estimating directly the probability of occurrence of a target species, it quantifies habitat suitability for its occurrence across the study area in question^16^, i.e. Madagascar. The predicted habitat suitability scores range from 0 to 1 (logistic output).

In order to assess the predictive power of the candidate explanatory variables and thus identify the most important ecological factors that determine habitat suitability at the species’ wintering grounds, we assessed the explanatory information in each variable when used in isolation and the information lost when that variable is omitted from a given model (i.e. training gain).

As a measure of the overall model predictive power, MaxEnt uses the Area Under the Curve (AUC score^17^). The AUC score ranges from 0.5, for models that predict no better than random, to 1.0, for models with perfect predictive power. However, considering the criticism against its use as a metric of model accuracy ^see 18, 19^, especially in cases where the spatial extent of the study area is large as in our case^18^, we assessed the statistical significance of the AUC scores of the resulting 100 models by contrasting them with the resulting values derived from null models following the recommended methodology in^20^.

Modeling future habitat suitability

We were interested in predicting future habitat suitability based on different climate change scenarios. To this end, we used the latest Global Circulation Models (GCMs) of the fifth phase of the Coupled Model Intercomparison Project (CMIP5; http://cmip-pcmdi.llnl.gov/cmip5/). GCMs used for forecasting climate change are generated based on scenarios concerning emissions of pollutants, future climate and environmental conditions, as well as socioeconomic changes. CMIP5 considers four scenarios, known as Representative Concentration Pathways (RCPs), of which we chose the two extreme ones, the low emissions scenario (RCP 2.6) and the high emissions scenario (RCP 8.5) to model future habitat suitability. RCP 8.5 presumes that no policy changes will be made to reduce emissions and thus CO2 concentrations will triple by 2100, reaching ca 1370ppm^21^. On the other hand, RCP2.6 presumes ambitious greenhouse gas emissions reductions (e.g. declining use of oil, increased bio-energy production), so CO2 concentrations will peak in 2050 and then decline to 400ppm by 2100^21^.

Among the available GCMs we used future climate data based on the HadGEM2-ES model^22^. In particular, we downloaded monthly precipitation and average monthly maximum temperature data for Madagascar for 2050 and 2070 (Worldclim, http://worldclim.org/). Then, as in the present model, we estimated the mean maximum temperature, the maximum and minimum precipitation for the period November – April for 2050 and 2070. We used these future climate variables instead of the current ones to produce future habitat suitability maps, while keeping the remaining variables as in the present model.

In total, we generated four future habitat suitability projections (2 scenarios x 2 years; hereafter, “**rcp2.6_2050 model**”, “**rcp2.6_2070 model**”, “**rcp8.5_2050 model**”, “**rcp8.5_2070 model**”) for Eleonora’s falcon in Madagascar and compared them to the present model.

**Table S1.** List of the tagged Eleonora’s falcons included in this study. The features of each individual, i.e. age, sex, origin, and the wintering event(s) tracked, as well as the device type used and the amount of data (number of fixes) retrieved are provided.

| ***Tag ID*** | ***Wintering period*** | ***Age*** | ***Sex*** | ***Data collection method*** | ***Country*** | ***Original dataset*** | | ***Modeling dataset*** | |
| --- | --- | --- | --- | --- | --- | --- | --- | --- | --- |
| 96574 | 10/11/2009 - 14/4/2010 | adult | female | 9.5g Argos PTT, Least squares, 10hON 48hOFF | Croatia (Svetac) | 310 | | 262 | |
| 96573 | 16/11/2009 - 4/4/2010 | adult | female |  |  | 254 | | 211 | |
|  | 28/11/2010 - 21/1/2011  (end of transmission) |  |  |  |  | 18 | | - | |
| 113739 | 19/11/2013 - 7/3/2014  (end of transmission) | adult | female | 5g Argos PTT, Kalman, 10hON 48hOFF | Cyprus (Akrotiri) | 195 | | 165 | |
| 113745 | 21/11/2013 - 21/3/2014  (end of transmission) | adult | female |  |  | 233 | | 194 | |
| 94118 | 14/11/2009 - 8/4/2010 | adult | female | 9.5g Argos PTT, Least squares, 6hON 70hOFF | Greece (Cyclades) | 73 | | 57 | |
| 94119 | 5/11/2009 - 11/4/2010 | adult | female |  |  | 246 | | 185 | |
| 94120 | 16/12/2009 - 13/4/2010 | juvenile | male |  |  | 167 | | 131 | |
| 94121 | 1/12/2009 - 6/5/2010 | juvenile | - |  |  | 349 | | 254 | |
| 40532 | 14/11/2003 - 19/4/2004 | adult | female | 18g Argos PTT, Least squares, 10hON 48hOFF | Italy (Sardinia) | 115 | | 99 | |
| 40536 | 20/1/2004 - 28/1/2004  (end of transmission) | juvenile | - | 18g Argos PTT, Least squares, 6hON 16hOFF |  | 8 | | - | |
| 49886 | 16/11/2004 - 26/3/2005 | adult | female |  |  | 227 | | 194 | |
| 49887 | 18/11/2004 - 20/4/2005 | adult | female |  |  | 231 | | 207 | |
| 49889 | 18/12/2005 - 8/1/2006  (end of transmission) | juvenile | female | 12g Argos PTT, Least squares, 10hON 48hOFF |  | 63 | | 30 (excluded due to low sample size) | |
| 49890 | 3/3/2005 - 19/4/2005 | adult | female |  |  | 56 | | 42 | |
| 49891 | 22/12/2005 - 6/5/2006 | juvenile | male |  |  | 311 | | 260 | |
| 80399 | 9/11/2008 - 6/4/2009 | adult | female | 9.5g Argos PTT, Least squares, 12hON 58hOFF | Spain (Balearics) | 311 | | 236 | |
|  | 11/11/2009 - 11/4/2010 | adult | female |  |  | 368 | | 279 | |
| 80400 | 16/11/2008 - 10/4/2009 | adult | male |  |  | 280 | | 206 | |
| 80402 | 15/11/2008 - 11/4/2009 | adult | female |  | Spain (Columbretes) | 313 | | 227 | |
|  | 14/11/2009 - 12/4/2010 |  |  |  |  | 337 | | 268 | |
| 92532 | 10/12/2010 - 24/4/2011 | juvenile | male |  |  | 201 | | 172 | |
| 1011 | 7/11/2012 - 11/4/2013 | adult | female | GPS | Spain (Canary islands) | 208 | | 173 | |
|  | 7/11/2013 - 5/4/2014 |  |  |  |  | 196 | | 159 | |
| 1012 | 13/11/2012 - 16/4/2013 | adult | male |  |  | 200 | | 163 | |
|  | 16/11/2013 - 18/4/2014 |  |  |  |  | 196 | | 163 | |
| 1013 | 10/11/2012 - 10/4/2013 | adult | male |  |  | 200 | | 152 | |
|  | 16/11/2013 - 10/4/2014 |  |  |  |  | 187 | | 151 | |
| 1014 | 18/11/2013 - 10/4/2014 | adult | female |  |  | 212 | | 167 | |
|  | 18/11/2013 - 10/4/2014 |  |  |  |  | 192 | | 154 | |
| **TOTAL** | | | | | | | **6,257** | | **4,967** |

**Table S2.** Explanatory variables used for modeling current habitat suitability.

| ***Variable*** | ***Acronym*** | ***Comments*** | ***Source*** |
| --- | --- | --- | --- |
| ***Topography*** | | | |
| Elevation (m) | elev |  | Worldclim, http://worldclim.org/ |
| Terrain slope (degrees) | slope |  | derived from elevation |
| Topographical roughness | roughn | Elevation difference within a 3x3 moving window, log-transformed ^1^ | derived from elevation |
| ***Climate regime*** | | | |
| Maximum precipitation (mm), for the period November – April (maximum of monthly values) | maxprec |  | Worldclim, http://worldclim.org/ |
| Minimum precipitation (mm), for the period November – April (minimum of monthly values) | minprec |  | Worldclim, http://worldclim.org/ |
| Mean maximum temperature (°C), for the period November - April (average of maximum monthly values) | meantemp |  | Worldclim, http://worldclim.org/ |
| ***Proximity to water*** | | | |
| Distance to water (classes) | distwater | 11 = water , 10 = 0-1km, 9 = 1-2km, 8 = 2-3km, 7=3-4km, 6=4-5km, 5=5-6km, 4=6-7km, 3=7-8km, 2=8-9km, 1=9-10km, 0 > 10km | derived from the hydrological network of the Digital Chart of the world, http://data.geocomm.com/readme/dcw/dcw.html |
| ***Vegetation composition and phenology*** | | | |
| mean NDVI, for the period November - April starting from November 2003 until April 2014 | meanndvi |  | MODIS monthly product MOD13A3, 1km resolution, https://lpdaac.usgs.gov/data_access/daac2disk |
| standard deviation of NDVI, for the period November - April starting from November 2003 until April 2014 | sdndvi |  | derived from meanndvi |
| percentage of vegetation classes in a 1km^2^ square grid cell | vegX | veg2= Bare Soil/Rock  veg4 = Cultivation  veg5 = Western dry forest  veg 6 = Plateau grassland-wooded grassland mosaic  veg 7 = Wooded grassland - bushland  veg 13 = Wetlands  veg 14 = Humid forest  veg 16= Degraded humid forest  Only those vegetation classes that occur in grid cells overlapping the original data were used, but for vegetation class 1 “Water” which has been already incorporated in the variable "distwater" | Vegetation map of Madagascar, http://www.vegmad.org/ |

**Appendix 2. Results**


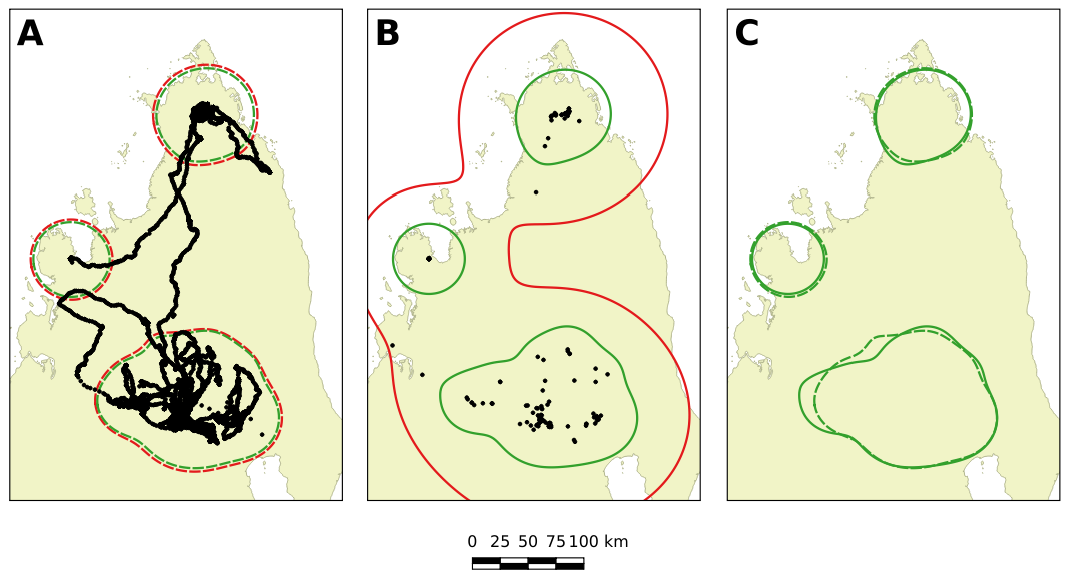


**Figure S1.** Exploratory analyses to validate the representation of the falcon trajectory after resampling. A) The original high resolution trajectory (5-min fix interval) of a single wintering event (one individual / year) is shown as black dots while the dashed polygons represent the home range (95%-KDE) obtained by both “h-ref” (red) and ad hoc (green) methods. B) Same kernel estimation as in A) by using the resampled dataset (4 random fixes / day). The “h-ref” method (red) in this case clearly overestimates the area used. C) Overlap between the home ranges (95%-KDE) estimated by ad hoc method by using the original high resolution dataset (dashed polygons) and the resampled dataset (solid polygons), UDOI = 1.85. Maps were created with ArcGIS v10.1 ([www.arcgis.com](http://www.arcgis.com/)).

| 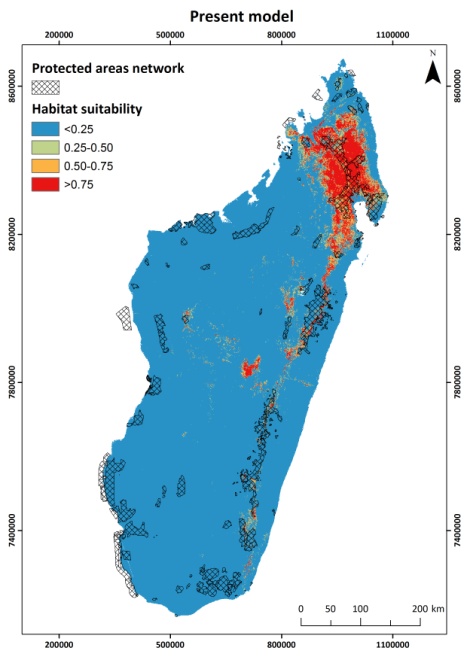 | |
| --- | --- |
| 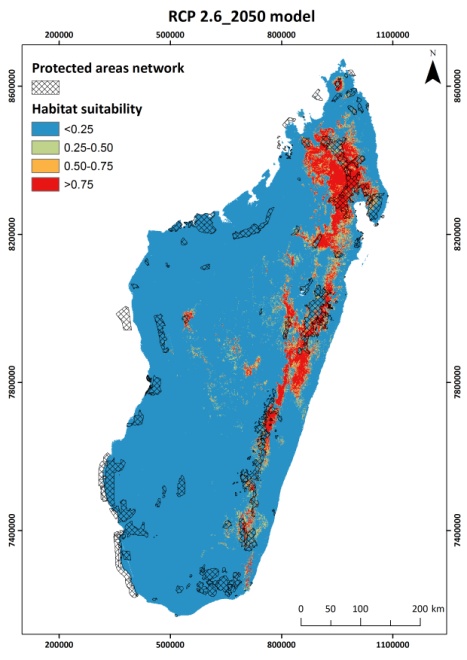 | 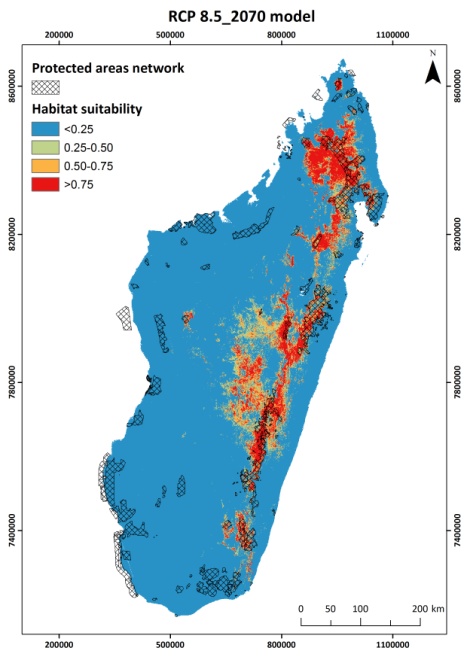 |
| 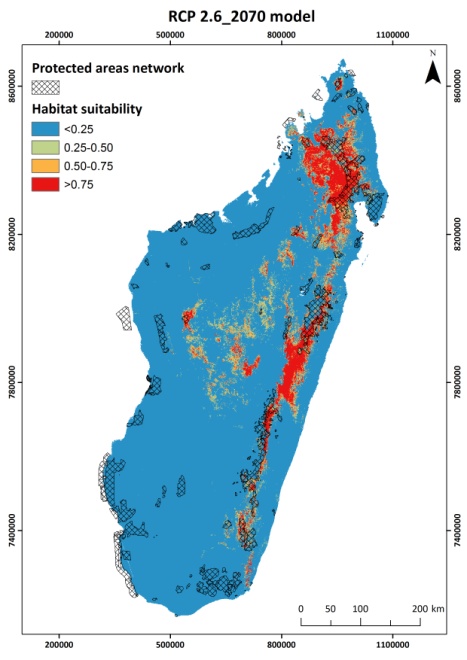 | 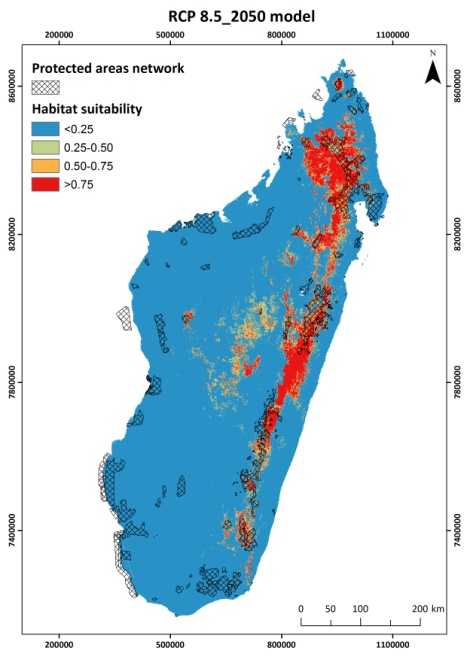 |
|  |  |

**Figure S2.** Predicted habitat suitability (average of 100 models) according to current environmental conditions (present model) and future climate scenarios (RCP2.6 and RCP8.5) for 2050 and 2070 for Eleonora’s falcon, based on satellite telemetry data of 17 adult falcons originating from colonies spanning from the westernmost (Canaries) to the easternmost (Cyprus) breeding range, relative to the protected areas network of Madagascar^23^. The percentage of overalp between highly suitable areas (i.e. with a habitat suitability score ≥ 0.75) and the existing protected areas network under present environmental conditions is 21% and expected to reach 22% on average under future climate scenarios (S.D. = 2%). Maps were created with ArcGIS v10.1 ([www.arcgis.com](http://www.arcgis.com/)).

**Table S3**. Home range overlap analysis results based on 95% Kernel Density Estimation (KDE) of 23 Eleonora’s falcons overwintering in Madagascar analyzed using the Utilization Distribution Overlap Index (UDOI). BA: Balearics (Spain) CA: Canary islands (Spain), CO: Columbretes (Spain), CR: Svetac (Croatia), CY: Akrotiri (Cyprus), GR: Cyclades (Greece), IT: Sardinia (Italy), AD: adults, JUV: juveniles

| **Group** | **Average UDOI values** | **Group** | **Average UDOI values** | |
| --- | --- | --- | --- | --- |
| BA-BA | 0.021 | CY-CO | 0.000 |  |
| BA-CO | 0.022 | CY-CR | 0.115 |  |
| BA-CR | 0.011 | CY-CY | 0.000 |  |
| BA-GR | 0.112 | CY-GR | 0.017 |  |
| CA-BA | 0.020 | CY-IT | 0.009 |  |
| CA-CA | 0.035 | GR-CR | 0.011 |  |
| CA-CO | 0.008 | GR-GR | 0.029 |  |
| CA-CR | 0.013 | IT-BA | 0.079 |  |
| CA-CY | 0.004 | IT-CO | 0.024 |  |
| CA-GR | 0.012 | IT-CR | 0.027 |  |
| CA-IT | 0.038 | IT-GR | 0.028 |  |
| CO-CO | 0.000 | IT-IT | 0.065 |  |
| CO-CR | 0.016 | AD-AD | 0.041 |  |
| CO-GR | 0.009 | AD-JUV | 0.016 |  |
| CR-CR | 0.000 | JUV-JUV | 0.009 |  |
| CY-BA | 0.003 |  |  |  |

**Table S4.** Extent of highly suitable areas (i.e., areas receiving a habitat suitability score greater than or equal to 0.75) based on the MaxEnt models produced under current and future climatic conditions.

| **Model** | **Year** | **Area**  **(sq.km.)** | **% area relative to the extent of the country** | **% area difference from present model** |
| --- | --- | --- | --- | --- |
| Present | - | 28,346 | 4.83 | - |
| RCP 2.6 | 2050 | 40,365 | 6.88 | 42.40 |
|  | 2070 | 38,697 | 6.59 | 36.52 |
| RCP 8.5 | 2050 | 44,710 | 6.88 | 57.73 |
|  | 2070 | 42,573 | 7.62 | 50.19 |

**Table S5**. Overlap of the existing protected areas network in Madagascar with the highly suitable habitat for Eleonora’s falcon both under present and future climatic conditions (i.e., areas with a habitat suitability score >0.75).

| **Model** | **Areas with habitat suitability score >0.75 (sq.km.)** | **Overlap relative to the extent of the protected areas network (%)** | |
| --- | --- | --- | --- |
| present | 28,346 | | 11.66 |
| RCP2.6_2050 | 40,365 | | 16.95 |
| RCP2.6_2070 | 38,697 | | 15.24 |
| RCP8.5_2050 | 44,710 | | 19.07 |
| RCP8.5_2070 | 42,573 | | 20.72 |

**References**

1. Gschweng, M., Kalko, E.K.V., Berthold, P., Fielder, W. & Fahr, J. Multi-temporal distribution modelling with satellite tracking data: predicting responses of a long-distance migrant to changing environmental conditions. *J. Appl. Ecol.* **49,** 803 – 813 (2012).

2. Mellone, U., López-López, P., Limiñana, R. & Urios, V. Wintering habitats of Eleonora’s Falcons *Falco eleonorae* in Madagascar. *Bird Study* **59,** 29 – 36 (2012).

3. Kassara, C., Fric, J. & Sfenthourakis, S. Distribution modeling of Eleonora’s Falcon *Falco eleonorae* Géné, 1839 occurrence in its wintering grounds: a niche-based approach with satellite telemetry data. *Bird Conserv. Int.* **24,** 100 – 113 (2014).

4. Viana, D.S., Gangoso, L., Bouten, W. & Figuerola, J. Overseas seed dispersal by migratory birds. *Proc. R. Soc. London Ser. B* **283,** 20152406 (2016).

5. Buy, R. & Gschweng, M. Opportunistic nocturnal hunting by Eleonora’s falcon *Falco eleonorae* on their breeding and non-breeding grounds. *Acta Ornithol.* **52** (2017).

6. Dormann, C.F. *et al.* Methods to account for spatial autocorrelation in the analysis of species distributional data: a review. *Ecography* **30,** 609–628 (2007).

7. Kramer-Schadt, S. *et al.* The importance of correcting for sampling bias in MaxEnt species distribution models. *Divers. Distrib.* **19,** 1366 – 1379 (2013).

8. Worton, B.J. Kernel methods for estimating the utilization distribution in home‐range studies. *Ecology* **70,** 164-168 (1989).

9. Walter, W.D., Fischer, J.W., Baruch-Mordo, S. & VerCauteren, K.C. What is the proper method to delineate home range of an animal using today’s advanced GPS telemetry systems: the initial step. USDA National Wildlife Research Center - Staff Publications. Paper 137 (2013).

10. Fieberg, J. & Kochanny, C.O. Quantifying home-range overlap: the importance of the utilization distribution. *J. Wildl. Manage.* **69,** 1346-1359 (2005).

11. Calenge, C. The package “adehabitat” for the R software: a tool for the analysis of space and habitat use by animals. *Ecol. Modell.* **197,** 516-519 (2006).

12. Dormann, C.F. *et al*. Collinearity: a review of methods to deal with it and a simulation study evaluating their performance. *Ecography* **35,** 27–46 (2013).

13. Edrén, S.M.C., Wisz, M.S., Teilmann, J., Dietz, R. & Söderkvist, J. Modelling spatial patterns in harbour porpoise satellite telemetry data using maximum entropy. *Ecography* **33,** 698 – 708 (2010).

14. Fielding, A.H. & Bell, J.F. A review of methods for the assessment of prediction errors in conservation presence/absence models. *Environ. Conserv.* **24,** 38 – 49 (1997).

15. Elith, J. *et al*. A statistical explanation of MaxEnt for ecologists. *Divers. Distrib.* **17,** 43 – 57 (2011).

16. Phillips, S.J., Anderson, R.P. & Schapire, R.E. Maximum entropy modeling of species geographic distributions. *Ecol. Modell.* **190,** 231 – 259 (2006).

17. Hanley, J.A. & McNeil, B.J. The meaning and use of the Area under a Receiver Operating Characteristic (ROC) Curve. *Radiology* **143,** 29 - 36 (1982).

18. Lobo, J.M., Jiménez-Valverde, A. & Real, R. AUC: a misleading measure of the performance of predictive distribution models. *Glob. Ecol. Biogeogr.* **17,** 145 – 151 (2008).

19. Phillips, S.J. & Dudík, M. Modeling of species distributions with Maxent: new extensions and a comprehensive evaluation. *Ecography* **31,** 161 – 175 (2008).

20. Raes, N. & ter Steege, H. A null-model for significance testing of presence-only species distribution models. *Ecography* **30,** 727 – 736 (2007).

21. Van Vuuren, D.P. *et al*. The representative concentration pathways: an overview. *Clim. Change* **109,** 5 – 31 (2011).

22. Collins, W.J. *et al.* Evaluation of the HadGEM2 model. Met Office Hadley Centre, Exeter, UK. <http://www.metoffice.gov.uk/media/pdf/8/7/HCTN_74.pdf> (2008).

23. IUCN & UNEP-WCMC. The World Database on Protected Areas (WDPA) [www.protectedplanet.net](http://www.protectedplanet.net/) (2015).
